# Next-gen paediatric imaging: ultra-low dose chest photon-counting CT approaching chest radiograph doses

**DOI:** 10.1186/s41747-026-00783-2

**Published:** 2026-09-08

**Authors:** Ione Limantoro, Kwinten Torfs, Joke Binst, Marijke Proesmans, Luc Breysem, Hilde Bosmans, Michael Aertsen

**Affiliations:** 1 Department of Radiology, University Hospital of Leuven, Leuven, Belgium; 2 Department of Imaging and Pathology, Catholic University Leuven, Leuven, Belgium; 3 Department of Paediatrics, University Hospitals Leuven, Leuven, Belgium

**Keywords:** Computed tomography, Child, Photons, Radiation dosage, Thorax

## Abstract

**Objective:**

Photon-counting detector (PCD) CT may reduce radiation dose while maintaining image quality, which is particularly promising for children due to their radiosensitivity. This feasibility study investigated whether an ultra-low dose CT (ULDCT) chest protocol on PCD CT, with a dose comparable to chest radiographs, could be developed to detect gross lung abnormalities in children.

**Materials and methods:**

This ethics-approved retrospective study consisted of two parts: (1) development of a ULDCT chest protocol using an anthropomorphic phantom and (2) qualitative evaluation of these scans performed in 24 consecutive patients (< 16 years) for various indications. Two paediatric radiologists assessed diagnostic quality for six chest pathologies using a 3-point Likert scale. Radiation dose metrics were extracted from a dose management system, and effective doses (ED) were calculated using age-appropriate conversion factors. Statistical analysis included mainly descriptive calculations. Doses were compared with routine chest radiography.

**Results:**

The Lungman phantom was scanned using 5 different protocols. The optimal settings were identified as Sn100 kVp, 2 mAs and pitch 1.5, yielding an ED of 14 µSv. All paediatric scans were assessed as acceptable, but a preference for an increased dose protocol was given to rule out interstitial lung disease or bronchiectasis. The mean total ED from the localiser and ULDCT of the chest was 11.6 [IQR: 10.9–12.8] µSv compared to 8.2 [IQR: 6.2–13.6] µSv for standard chest radiography.

**Conclusion:**

Our PCD ULDCT chest protocol, approaching chest radiograph doses, is promising for (follow-up of) focal abnormalities and follow-up imaging of known chest pathology in children.

**Key Points:**

- **Question** Can the technique of photon-counting detector CT result in radiation dose reduction, while achieving adequate image quality?
- **Findings** An ultra-low dose CT chest protocol was developed with a comparable dose to a single chest radiograph.
- **Relevance statement** The ultra-low dose CT chest protocol is promising in the setting of (follow-up of known) focal chest abnormalities, long-term follow-up after congenital diaphragmatic hernia repair and follow-up of interstitial lung disease in children.

**Graphical Abstract:**

**Figure:**
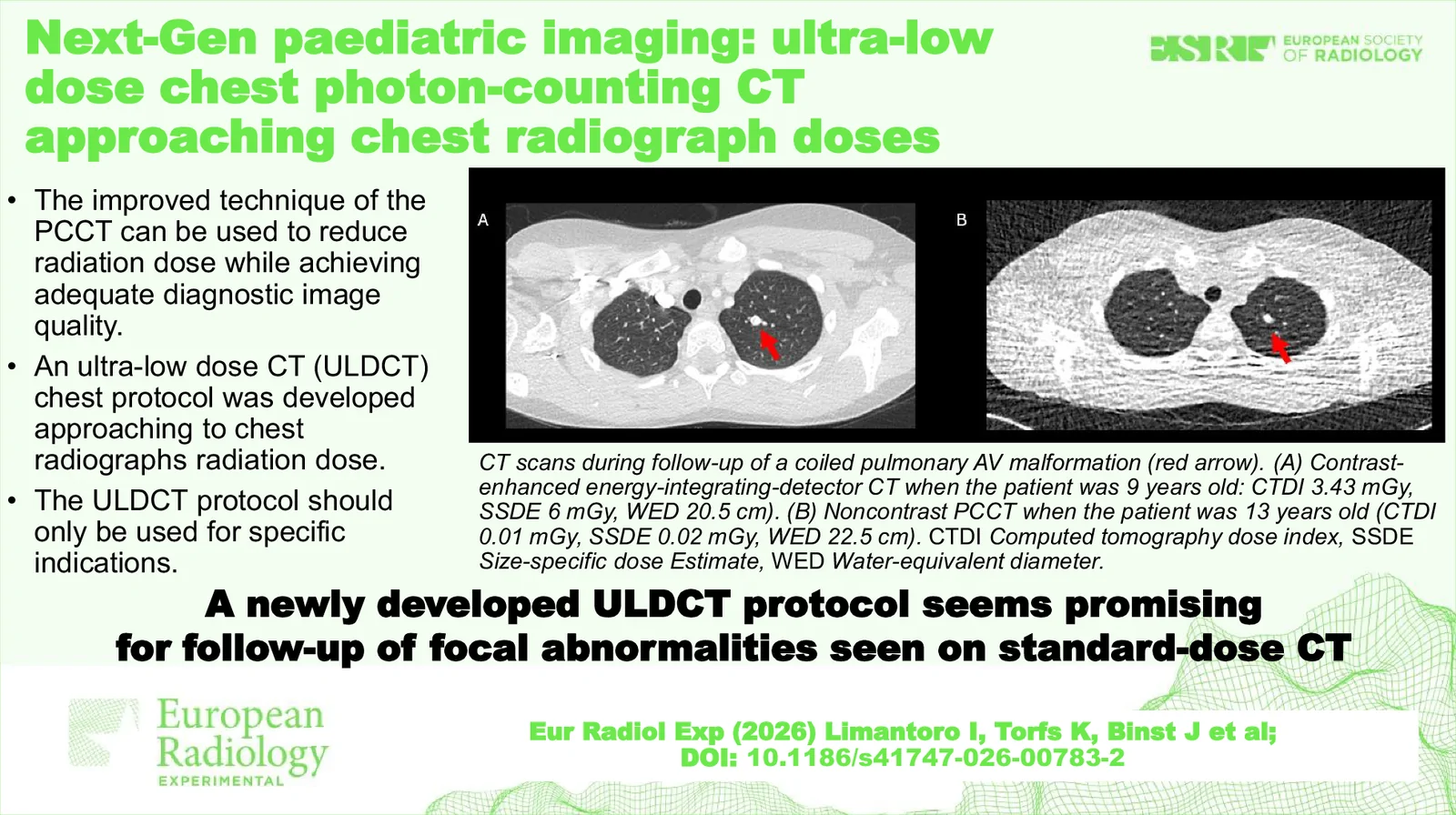

## Background

The use of CT scans in children has increased significantly over the past decades [1, 2]. Although CT often provides a more accurate evaluation than a radiograph, it is generally not the modality of choice due to its higher radiation dose. Therefore, several paediatric ultra-low-dose CT (ULDCT) protocols have been developed for different indications, including preoperative imaging of craniosynostosis, craniofacial anomalies or pectus excavatum, skeletal survey for suspected non-accidental injury or evaluation of ventriculoperitoneal shunts [3–7]. Especially in chest imaging, CT plays an important role, and many studies have focused on lowering the CT dose, for example, for the evaluation of cystic fibrosis, foreign body aspiration and preoperative assessment of pectus excavatum [8–10]. The development of new CT dose-reduction strategies remains crucial. The most recent technical development in CT technology is the photon-counting detector (PCD) CT scanner. This scanner has the potential to lower x-ray doses, as shown in selected studies [11–16]. Conventional energy-integrating CT (EID-CT) uses scintillators, which convert x-rays into visible light. During this process, septa are required to prevent light from scattering between detector pixels. With PCD CT, the detector directly converts x-rays into electrical signal, the classical septa are no longer required, and this property has been used to increase spatial resolution. In addition, *via* the counting of photons, electronic noise does not enter the signal. This is particularly promising for low-dose acquisitions [13, 14]. Based on reduced noise and improved spatial and contrast resolution at decreased radiation doses, paediatric imaging could potentially benefit from this new imaging technique [11, 16–18]. However, CT potentially offers better accuracy in detecting recurrence. This consideration led to the development of a ULDCT protocol using the PCD CT. The hypothesis of the present study is that a ULDCT chest protocol can be developed with radiation dose settings in terms of effective dose that are comparable to chest radiographs. This article describes the development of a ULDCT protocol for chest CT exams for the detection of gross and large pathologies in the lungs of children. The new protocol was then challenged in successive paediatric cases referred for various indications.

## Materials and methods

### Study design and patient selection

This IRB-approved retrospective feasibility study consisted of two parts: (I) development of a ULDCT chest protocol using phantom scans and (II) analysis of paediatric chest CT scans using this newly proposed ULDCT protocol. This study was approved by our ethical committee (IRB number S-68087, University Hospital of Leuven, Leuven, Belgium). Informed consent was waived.

### Lungman phantom

**Fig. 1:**
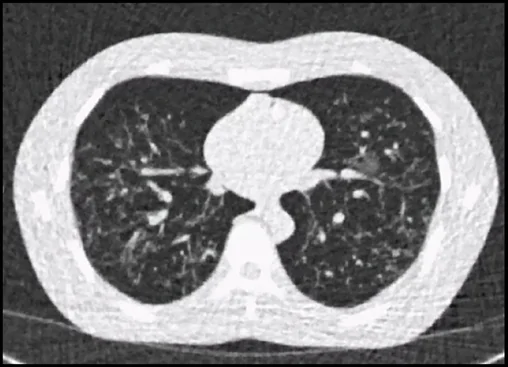
Scan performed on the Lungman phantom

Different strategies of implementing paediatric ULDCT were compared using an anthropomorphic chest phantom (Fig. 1). The anthropomorphic phantom was preferred over a smaller, pure polymethyl methacrylate phantom, as it more closely resembled anatomic structures. The multipurpose thorax phantom Lungman (Kyoto Kagaku Co., Japan) resembles the thorax of a patient in an arms-abducted position with a water equivalent diameter (WED) of 23 ± 6 cm as measured on CT and was developed for protocol optimisation in radiography and computed tomography. The phantom consists of several types of resin: urethane-based for soft tissue and epoxy-based for bone material. The thorax is completed with shoulders and ribs, a heart, a homogeneous abdomen block, and an air-filled lung cavity with pulmonary vessels and bronchi. In our Lungman set-up, there are anatomically accurate thoracic structures, but the phantom does not represent lung tissue.

#### I. Comparing strategies for ultra-low dose chest CT on phantom scans

**Acquisition and reconstruction settings**: All scans in this study were performed on a Siemens NAEOTOM Alpha photon-counting CT (Siemens Healthineers, Erlangen, Germany). The scout image was performed at 100 kVp with tin filtration, 21 mAs and reconstruction kernel Tr20u.

Ultra-low dose was defined as equal to or lower than doses associated with standard chest radiography exams. This was achieved with a tube voltage of 70 kVp or 100 kVp with added tin filtration, pitch of 1.5 or 3.2. a fixed lowest exposure of 1 or 2 mAs, gantry rotation time of 0.25 s, a z-coverage of 144 × 0.4 mm and a focal spot size of 0.8 × 1.2 mm. The usefulness of tube current modulation was explored at 70 kVp with CareDOSE 4D at IQ level 1.

Combinations of these acquisition settings resulted in five distinct dose-reduction strategies, as described in Table 1. Reconstruction settings were identical for each scan strategy with a field-of-view (FOV) of 350 mm, 512 × 512 matrix size, and a resulting pixel size of 0.68 mm (= FOV/matrix size). Monoenergetic images were reconstructed at 73 keV with reconstruction kernels Bl56 for lung and Br40 for soft tissue evaluation, both at an iterative strength of QIR 4, and slice thicknesses of 1 and 3 mm, respectively.

**Table 1:** Phantom scan parameters using different kVp, pitch and use of CareDOSE 4D

	Chest radiograph	Scout	ULDCT A	ULDCT B	ULDCT C	ULDCT D	ULDCT E
CareDOSE 4D	RX AEC	-	Off	Off	Off	Off	On
Voltage (kVp)	110	Sn100	Sn100	Sn100	70	70	70
Effective tube current (mAs)	1	21	2	1	2	1	5 ± 4
Pitch	-	-	1.5	3.2	1.5	2.4	1.5
DAP (dGy.cm²)	0.18						
CTDI_vol_ (mGy)	-	-	0.01	0.01	0.02	0.03	0.05 ± 0.05
DLP (mGy.cm)	-	0.11	0.54	0.55	0.92	1.31	2.00
ED (mSv)	0.008	0.003	0.014	0.014	0.023	0.033	0.050

*AEC* Automatic exposure control, *CTDI*_*vol*_ CT dose index, *DAP* Dose area product, *DLP* Dose length product, *ED* Effective dose, *ULDCT* Ultra-low dose CT

**Visual assessment**: The anthropomorphic phantom was then scanned with each strategy, and the subsequent images were subjectively assessed by one radiologist (M.A.), blinded to protocol settings, on a diagnostic workstation. Phantom scans were classified according to the radiologist’s preference in the assessment of diaphragmatic hernia recurrence and lung volume.

**Radiation dose**: Effective doses were derived from the dose length product (DLP) for each ULDCT scan using dose conversion factors that were validated within our institution. The DLP and computed tomography dose index volume (CTDI_vol_), referenced to a 32-cm phantom, were received from the scanner *via* the radiation dose structured report (RDSR), and then extracted by our institution’s dose management software (DOSE, Qaelum, NV, Belgium).

The target age for the ULDCT chest protocol was 8 years, and thus the effective dose for the phantom scans was calculated using the dose conversion factor associated with that age: 0.025 mSv/mGy.cm. The exam’s total effective dose combined both the scout view and CT scan series, as the contribution of the first cannot be neglected at such low dose levels.

For reference comparison, the effective dose of a one-view standard (anterioposterior or posterior-anterior) chest radiograph, taken with the same Lungman phantom, was determined from the dose area product (DAP) with a conversion factor of 0.0469 mSv/dGy.cm² for an 8- year-old.

#### II. Clinical implementation and validation of the ULDCT chest

After determining the optimal ULDCT protocol, it was implemented into clinical practice in January 2024. All non-contrast chest CT scans performed in children ≤ 16 years of age using the ULDCT protocol between January 2024 and May 2026 were included and analysed for radiation dose (as described above) and image quality. There were no exclusion criteria.

**Acquisition and reconstruction settings**: Protocol settings were copied from the optimal ULDCT strategy, as determined on the phantom. The technologist manually set the reconstruction FOV on the scout image.

**Visual assessment**: A visual grading assessment was conducted. All images were anonymised and no clinical information was provided. The scans were uploaded to a cloud-based database for radiology images (CMRAD, Collective Minds, Sweden), and diagnostic quality was assessed on the 1 mm thickness slices in lung window on a diagnostic workstation. Based on the indications for ULDCT scan, the examinations were categorised into six specific diagnostic tasks (Table 2): (1) rule out focal abnormalities (for example AVM, nodule), (2) rule out interstitial lung disease (ILD, clinically low likelihood), (3) are there bronchiectasis, (4) follow-up of a known focal abnormality on a previous CT (for example AVM, nodule, cyst), (5) long-term follow-up after congenital diaphragmatic hernia repair, (6) follow-up of ILD (deterioration/improvement). Two paediatric radiologists scored every ULDCT scan for these six diagnostic tasks using a 3-point Likert scale: (1) uncomfortable to answer the clinical question, an extra CT scan with standard dose should be performed immediately during the same session, (2) still comfortable to answer the clinical question but the next follow-up scan should be performed with a standard-dose CT scan, (3) comfortable to answer the clinical question, no additional standard-dose CT scan necessary. The median and interquartile range (IQR) for VGA scores are provided for each clinical indication, and results are shown in Table 2. Interobserver agreement is assessed using the absolute observer agreement percentage. In addition, the weighted Cohen’s kappa was calculated.

**Table 2:** Image quality assessment of ULDCT scans with a 3-point Likert scale

Indication	Image quality assessment Median (IQR 25–75%)	Number of scans performed for this indication
Rule out focal abnormalities (for example, AVM, nodule)	3.0 (2.5–3.0)	4
Rule out ILD (when clinical likelihood is low)	2.5 (2.0–3.0)	2
Are there bronchiectasis?	2.8 (2.5–3.0)	1
Follow-up chest CT with known focal abnormality (for example, AVM, nodule, cyst)	3.0 (3.0–3.0)	9
Long-term follow-up after congenital diaphragmatic hernia repair	3.0 (3.0–3.0)	3
FU of ILD (when the question is solely: deterioration/stable/improvement)	3.0 (3.0–3.0)	5

(1: Uncomfortable to answer the clinical question, an extra CT scan with standard dose should be performed immediately during the same session, 2: Still comfortable to answer the clinical question, but next follow-up scan should be performed with a normal dose CT scan, 3: Comfortable to answer the clinical question)

*AVM* Arteriovenous malformation, *CT* Computed tomography, *FU* Follow-up, *ILD* Interstitial lung disease, *ULDCT* Ultra-low-dose CT

**Radiation dose**: Similar to the phantom scans, the ULDCT effective doses were calculated from the DLP, retrieved from the dose management system (Dose, Qaelum NV, Belgium), through dose conversion factors. Age-specific conversion factors were applied: 0.036, 0.032, 0.028, 0.025 and 0.021 mSv/mGy.cm for age groups (0–1), (1–4), (4–8), (8–13) and (13–17) years. Patient size was expressed in terms of water equivalent diameter (WED) with an in-house built tool [19], averaged over 20 equidistant slices. Size-specific dose estimates (SSDE) were determined with the same toolbox.

In order to compare the dose of the patient ULDCT scan to chest radiograph doses of patients of a similar age and size, we collected all chest radiographs in children (< 18 years) acquired between January 2020 and May 2026. We included radiographs of children who also received a CT scan within an interval of 12 months, in order to have a WED of the patient based on the CT scan. Using this WED, a parameter for patient size, the patients could be matched to the ULDCT scan patients.

### Statistical analysis

Statistical calculations were performed with commercially available statistics software (Prism version 5.04, GraphPad). Similarity in age and WED distribution between the patient ULDCT and radiography group was assessed through a Kolmogorov–Smirnov test. The difference in effective dose was determined by a Wilcoxon–Mann–Whitney test. Further, values are reported as mean and standard deviation or median and interquartile range (IQR). For visual grading analysis, descriptive statistics are calculated in terms of (median – IQR).

## Results

### Comparing strategies for ultra-low dose chest CT on phantom scans

The radiation doses for the five distinct ULDCT implementation strategies are found in Table 1. Using tube current modulation resulted in a higher average exposure of 5 mAs, as the system could not modulate below 1mAs. Therefore, modulation did not provide any benefit at these ultra-low dose levels. Fixed exposure protocols used either 1 or 2 mAs and were thus preferred.

All ULDCT strategies were visually evaluated according to their quality to assess the recurrence of diaphragmatic hernia and lung volume. The optimal scan was chosen based on the lowest possible effective dose: ULDCT A (Table 1) with the following parameters: Sn100 kVp, 2 mAs, 1.5 pitch, 0.25 s gantry rotation time, 144 × 0.4 mm z-coverage, and a 0.8 × 1.2 mm focal spot. The associated effective dose was 0.014 mSv, or 1.8 times what was measured on standard chest radiography of the phantom.

### Clinical implementation and validation of the ULDCT chest

**Fig. 2:**
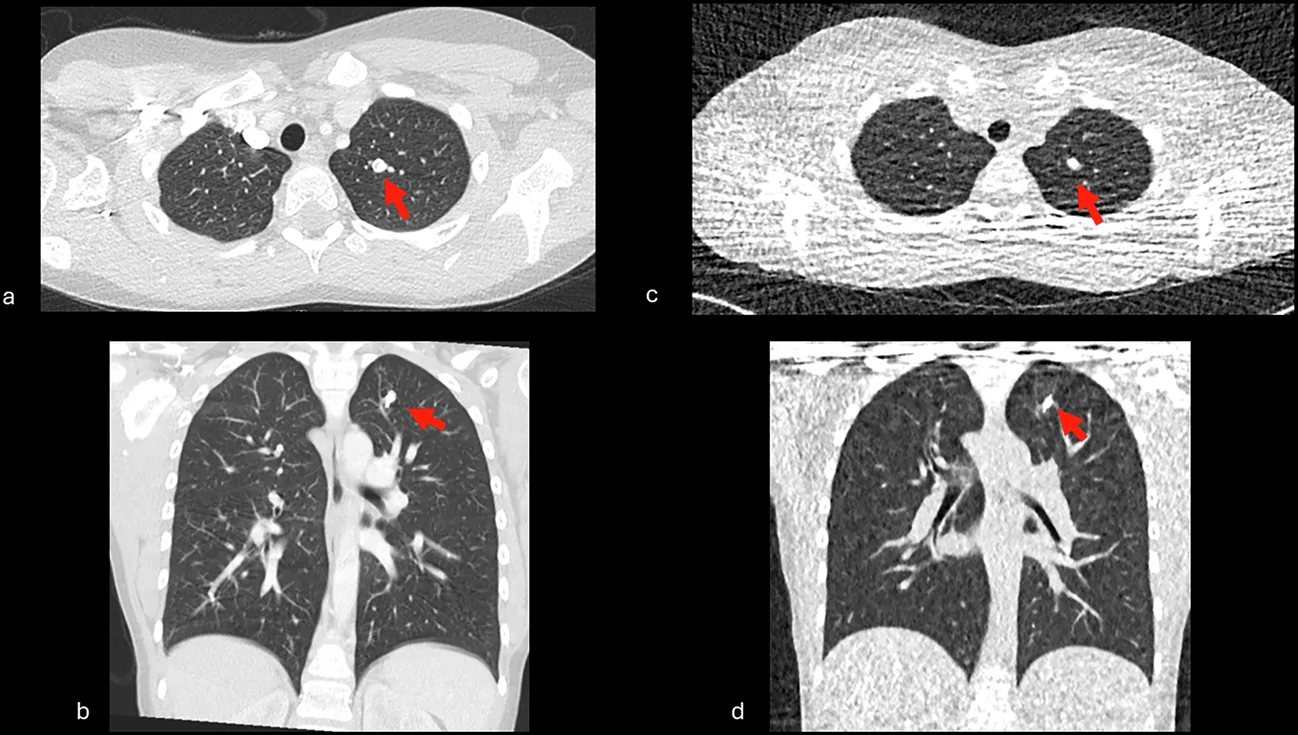
CT scans during follow-up of a patient known with a coiled pulmonary AV malformation in the left upper lobe (red arrow). Axial (**a**) and coronal (**b**) images obtained on energy-integrating-detector CT (with intravenous contrast) when the patient was 9 years old (CTDI 3.43 mGy, SSDE 6 mGy, WED 20.5 cm). Axial (**c**) and coronal (**d**) images obtained on PCCT using ULDCT protocol (without intravenous contrast) when the patient was 13 years old (CTDI 0.01 mGy, SSDE 0.02 mGy, WED 22.5 cm)

**Fig. 3:**
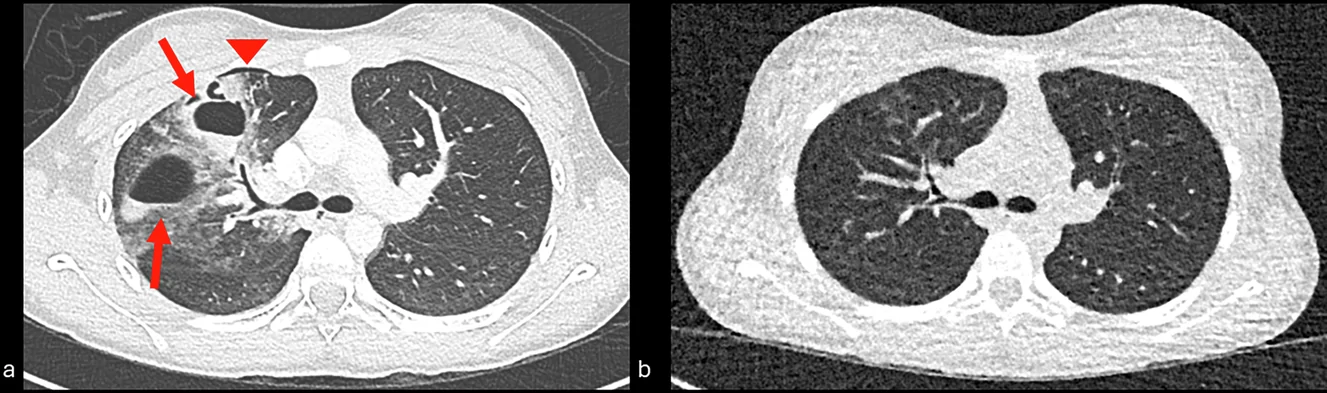
CT scan of a 12-year-old girl with posttraumatic pneumatocele. Normally, a follow-up CT scan is not indicated when asymptomatic. However, the pneumatologist needed to make sure there was no residual air leak because she was going on a diving trip by airplane. Axial (**a**) image obtained on energy-integrating-detector CT (with intravenous contrast) (CTDI 1.36 mGy, DLP 43 mGycm): pneumatocele (red arrow) and pneumothorax (arrowhead). Axial (**b**) image obtained on PCCT using ULDCT protocol (without intravenous contrast) when the patient was 13 years old (CTDI 0.01 mGy, DLP 1 mGycm): no residual pneumatocele or pneumothorax

In total, 24 ULDCT scans were performed in 20 patients over a 29-month time period using the ULDCT A protocol for different indications (Table 3). The different CT indications were grouped into 6 specific diagnostic tasks (Table 2). The majority of scans (9/24) were performed in the setting of follow-up of a known focal abnormality on a previous standard-dose CT. Median patient age was 10 years [IQR: 4–13], ranging from 4 months to 16 years, with a WED of 19 [IQR: 15–21] cm, ranging between 12 and 24 cm. Two example cases scanned with the selected ULDCT protocol are presented in Figs. 2 and 3.

**Table 3:** Indications for ULDCT chest protocol and corresponding ages of patients

Patient	Indication of CT scan	Age of patient	Previous CT (if yes, number of previous CT scans)
1	FU congenital diaphragmatic hernia: recurrence hernia? Tracheal abnormalities?	10 years	No
2	FU congenital diaphragmatic hernia: recurrence hernia? Tracheal abnormalities?	9 years	Yes (1)
3	Known systemic juvenile idiopathic arthritis: FU of known interstitial lung disease	2 years	Yes (3)
4	Cystic fibrosis: bronchiectasis? (technologist accidentally selected this protocol)	3 years	No
5	Suspicion systemic sclerosis with shortness of breath: rule out ILD.	14 years	No
6	Known Wilms tumour. FU non-specific nodules: growth?	5 years	Yes (3)
7	Known Rendu-Osler Weber: FU after coiled pulmonary AVM	13 years	Yes (4)
8	New diagnosis Rendu-Osler Weber: rule out pulmonary AVM	4 months	No
3	Known systemic juvenile idiopathic arthritis: FU of known interstitial lung disease, evolution nodule	2 years	Yes (4)
9	FU posttraumatic pneumatocele: residual pneumothorax/cele? Normally, no CT necessary, but the patient planned a trip by airplane to go diving, so important to know there was no residual air leak.	12 years	Yes (1)
10	Cantu syndrome. Rule out pulmonary AVM	5 years	No
11	Status after congenital diaphragmatic hernia repair. On radiograph abnormality: hernia recurrence or atelectasis?	15 years	No
12	Limited systemic sclerosis. ILD?	15 years	Yes (1)
13	FU echinococcus cyst.	11 years	Yes (4)
3	Known systemic juvenile idiopathic arthritis: FU of known interstitial lung disease	3 years	Yes (5)
14	New diagnosis Rendu-Osler Weber: rule out pulmonary AVM	7 years	No
13	FU echinococcus cyst.	11 years	Yes (5)
15	Status after lobectomy in the setting of bronchiectasis. FU: New bronchiectasis?	10 years	Yes (2)
16	FU tuberculosis infection with cavernous lesions	14 years	Yes (1)
17	Known chronic granulomatous disease, mycosis? Many recent CT scans.	11 years	Yes (6)
18	FU tuberculosis with atelectasis and granuloma	5 years	Yes (3)
19	New diagnosis Rendu-Osler Weber: rule out pulmonary AVM	13 years	No
20	FU CPAM	1 year	Yes (1)
3	Known systemic juvenile idiopathic arthritis: FU of known interstitial lung disease	4 years	Yes (6)

*AVM* Arteriovenous malformation, *CT* Computed tomography, *FU* Follow-up, *ILD* Interstitial lung disease, *ULDCT* Ultra-low-dose CT

All patient scans received a visual grading score indicating sufficient confidence to answer the intended clinical question without immediate repeat standard-dose CT. The results of the visual grading assessment are shown in Table 2, with a median score of 3.0 to rule out focal abnormalities, follow-up chest CT of known focal abnormalities, long-term follow-up after congenital diaphragmatic hernia repair or follow-up of interstitial lung disease (ILD) (when the question is solely: deterioration/stable/improvement). Ruling out ILD and bronchiectasis was scored with a median of 2.5 and 2.8, respectively. The absolute overall reader agreement was 79%. The weighted Cohen’s kappa was 0.365 (standard error = 0.09).

Regarding the chest radiographs, 791 paediatric chest radiographs were included in this study for dose distribution calculation. The median patient age was 9 years [IQR: 4–14], ranging from 4 days to 18 years, with a WED of 18 [IQR: 15–21] cm, ranging between 10 and 30 cm. Regardless of the large extent, the distributions of both age (*p* = 0.73) and WED (*p* = 0.76) did not differ significantly between the ULDCT and radiography group.

**Fig. 4:**
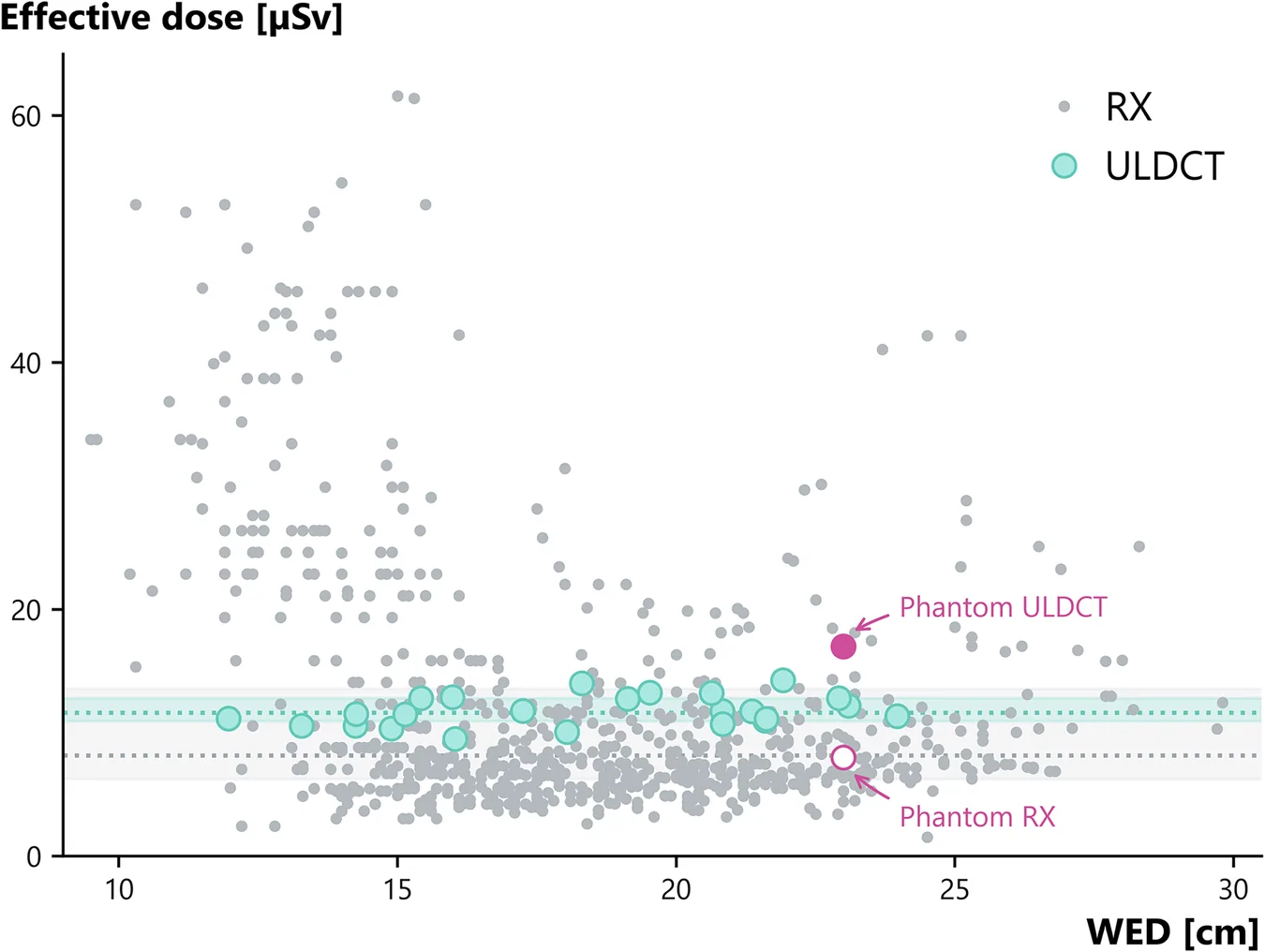
Effective dose per patient study of ULDCT and standard chest radiograph as a function of WED, showing that radiation doses of the ULDCT are comparable, or even lower for lower WEDs, than in standard chest radiographs

The median total effective dose for ULDCT was 11.6 [IQR: 10.9–12.8] µSv, split between 1.7 [IQR: 1.5–1.8] µSv from the scout image and 9.9 [IQR: 9.4–11.1] µSv from the CT scan series. In comparison, the median total effective dose for a single AP chest radiograph was 8.2 [IQR: 6.2–13.6] µSv (*p* = 0.007), also seen in Fig. 4.

An overview of these doses and other parameters (CTDI_vol_, DLP, WED and SSDE) is given in Table 4 and Fig. 4.

**Table 4:** Radiation doses of ULDCT and chest radiographs (RX) for the same WED values within 11 to 23 cm

	ULDCT	RX
Number of exams	24	791
Age (years)	10 [IQR 4–13]	9 [IQR 4–14]
WED (cm)	19 [IQR 15–21]	18 [IQR 15–21]
Mean ED (µSv)	11.8 ± 1.2	13 ± 11
Median ED (µSv)	11.6 [10.9–12.8]	8.2 [6.2–13.6]
*CTDI*_*vol*_ *(mGy)*	0.01	/
*DLP (mGy.cm)*	0.4 ± 0.1	/
*SSDE (mGy)*	0.019 ± 0.002	/
*DAP (dGy.cm²)*	/	0.17 [IQR: 0.11–0.27] 0.3 ± 1.5

Mean values and standard deviations are given for age, WED, CTDI_vol_, DLP, SSDE and DAP and ED

*CTDI*_*vol*_ CT dose index, *DAP* Dose area product, *DLP* Dose length product, *ED* Effective dose, *RX* Radiograph, *SSDE* Size-specific dose estimate, *ULDCT* Ultra-low dose CT, *WED* Water equivalent diameter

## Discussion

Using the Lungman chest phantom, PCD CT parameters were optimised to achieve radiation doses comparable to those in standard chest radiography, while maintaining sufficient image quality to assess congenital diaphragmatic hernia recurrence. Our preliminary results using this ULDCT protocol in 24 paediatric cases suggest that new CT exposure settings with doses approaching the level of chest radiographs could potentially be used to assess (follow-up of known) focal abnormalities, long-term follow-up after congenital diaphragmatic hernia repair or follow-up of ILD.

The introduction of the PCD CT has already allowed the development of new protocols for various applications, resulting in dose reductions while maintaining diagnostic image quality [13–16]. This is especially promising for paediatric imaging, where structures are smaller and patients are more sensitive to radiation. Nonetheless, this new technology has primarily focused on maintaining dose levels while gaining higher resolution. In this study, however, the capabilities of the PCD CT were used to significantly reduce the radiation dose while maintaining the necessary diagnostic image quality for detecting gross or large abnormalities to answer the specific clinical questions, taking into account the decreased image quality compared to regular dose CT.

To our knowledge, our ULDCT scan protocol has a lower dose compared to other PCD CT studies published earlier. Previously, Villanueva-Meyer et al already described an ultra-low dose protocol for specific indications such as foreign body evaluation (CTDI 0.20 ± 0.08 mGy, SSDE 0.4 ± 0.1 mGy) [20]. Recently, a chest ULDCT using PCD CT in children was developed, which was suitable to assess lung parenchyma (effective dose of 190 ± 70 µSv, CTDI_vol_ 0.21 ± 0.08 mGy, SSDE 0.45 ± 0.14 mGy) [16]. The ULDCT protocol described in our study resulted in an even lower dose (CTDI_vol_ 0.01 mGy, effective dose 11.8 ± 1.2 µSv, SSDE 0.019 ± 0.002 mGy), comparable to standard chest radiography. The dose from the scout view was included, as the contribution of the scout view becomes non-negligible at such low dose levels.

There are many clinical scenarios for which international follow-up guidelines are not yet established, such as the surveillance of congenital diaphragmatic hernia patients or screening for pulmonary arteriovenous malformations [21, 22]. This is often due to the challenging balance between optimal imaging of the lungs and minimising radiation exposure in children. At the same time, the cumulative dose paradigm emphasises that lifetime doses for one patient can increase due to recurrent imaging [23, 24]. Especially because the threshold for clinicians to request a chest CT has also become lower, ULDCT protocols can help mitigate this paradigm. This means that even an image with reduced image quality can be sufficient for specific tasks. For patients who are expected to undergo multiple CT scans over time—such as follow-up of pulmonary arteriovenous malformations—alternating between standard and ultra-low dose CT scans potentially helps minimise their overall radiation exposure. For example, when a nodule is seen on a standard CT scan and the only question is volume evaluation of a nodule, a ULDCT would be an option. In our institution, the ULDCT protocol was primarily used to rule out or monitor focal abnormalities (cysts, nodules, pulmonary AVM). It was also utilised to evaluate ILD deterioration or improvement. Choosing the ULDCT protocol should always be discussed with the clinician to assess the therapeutic implications, and only if necessary, a standard-dose CT can be added during the same session. As the dose of the ULDCT was comparable to radiography, this approach was considered acceptable. In none of the presented cases was this necessary. However, it is important to recognise that our ULDCT scan protocol does not provide the same level of diagnostic information as standard CT. The visual grading assessment showed that the protocol is less appreciated for ruling out ILD and bronchiectasis without prior imaging for comparison.

This single-centre study was intended as a feasibility study, with the small and heterogeneous patient group in this single-centre study as an important limitation. Since there were no specific criteria to scan patients using the ULDCT protocol, but it was rather case-based, the selection of patients was biased. Therefore, we provided an overview of all patients with the clinical question and also grouped the indications into specific diagnostic tasks. Another limitation was that the ULDCT was not compared to a standard-dose chest CT in the same patient at the same time to compare diagnostic performance, but this would violate the ALARA (as low as reasonably achievable) principle. However, this was not the goal of this feasibility study. The phantom evaluation was limited due to assessment by a single reader and a lack of objective evaluative criteria. However, the evaluation of the patient’s ULDCT scans is clinically more important and was done by two radiologists. An objective assessment of the image quality was technically not possible due to the limited lung parenchyma in small patients for global noise level assessment and would be of poor statistical value in this small group.

Based on phantom testing, we developed a ULDCT chest protocol using PCD CT that approached the radiation dose of a single chest radiograph. This feasibility study shows promising results for the use of ULDCT scans for (follow-up of known) focal abnormalities, long-term follow-up after congenital diaphragmatic hernia repair or follow-up of ILD. More studies with larger paediatric populations are needed to evaluate further diagnostic performance of this ULDCT chest scan, for example, in the setting of chronic airway and interstitial disease.

## Data Availability

Data are available upon request.

## Abbreviations

CT: Computed tomography
CTDI_vol_: CT dose index volume
DAP: Dose area product
DLP: Dose length product
ED: Effective dose
FOV: Field-of-view
kVp: Peak kilovoltage
mAs: Milliampere-seconds
PCD CT: Photon-counting detector CT
SSDE: Size-specific dose estimate
ULDCT: Ultra-low-dose CT
WED: Water equivalent diameter
